# Following Pickwick’s Advice

**Published:** 1881-10

**Authors:** 


### FOLLOWING PICKWICK'S ADVICE!

"'Slumkey, forever!' shouted Mr. Pickwick, taking off his hat. * * * *

"'Who is Slumkey?' whispered Mr. Tupman.

"'I don't know,' replied Mr. Pickwick, in same tone. 'Hush! Don't ask any questions. It is always best on these occasions to do what the mob do.'

"'But, suppose there are two mobs!' suggested Mr. Tupman.

"'*Shout with the largest,*' replied Mr. Pickwick."

Many *homœopathists*, having axes to grind, find Mr. Pickwick's advice good just now. Hence the outcry against the Internationals. Persons of small mind and mean disposition will sink truth any day for popularity and—cash.

